# Supplementary material for: Comprehensive analysis of microbial content in whole-genome sequencing samples from The Cancer Genome Atlas project
Source: bioRxiv. 2024 Aug 19:2024.05.24.595788. Preprint. [Version 3] doi: 10.1101/2024.05.24.595788 (PMC11275966; doi:10.1101/2024.05.24.595788)
Supplement: Supplement 7 [file media-7.docx]

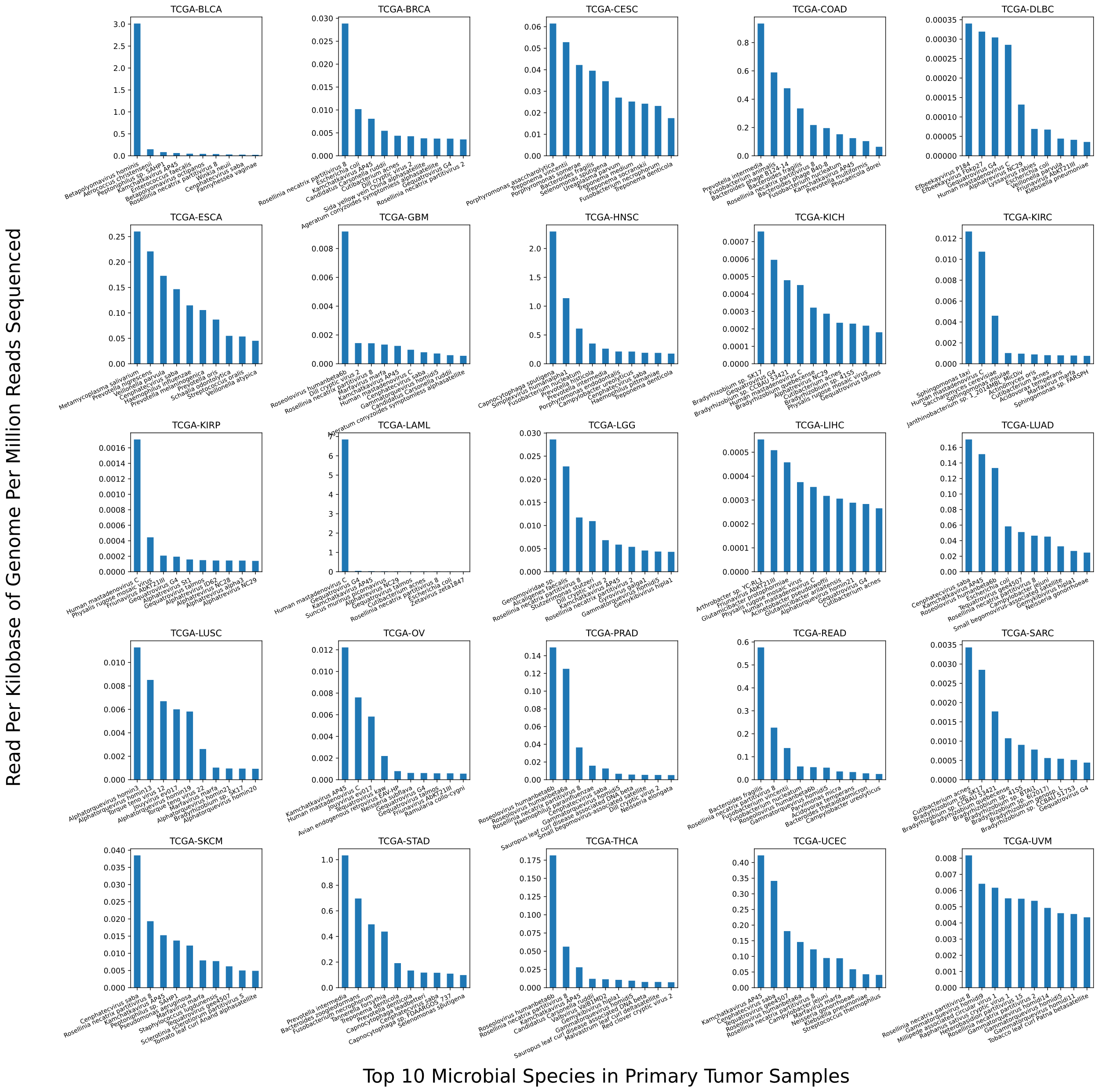


**Supplementary Figure S1.** Normalized counts of the top 10 microbial species in primary tumor samples for each of 25 cancer types. The X-axis shows the species names, sorted by maximum normalized counts, measured as reads per kilobase of genome per million reads sequenced.
