# Supplementary material for: Comprehensive analysis of microbial content in whole-genome sequencing samples from The Cancer Genome Atlas project
Source: bioRxiv. 2024 Aug 19:2024.05.24.595788. Preprint. [Version 3] doi: 10.1101/2024.05.24.595788 (PMC11275966; doi:10.1101/2024.05.24.595788)
Supplement: Supplement 8 [file NIHPP2024.05.24.595788v3-supplement-8.pdf]

bioRxiv preprint doi: https://doi.org/10.1101/2024.05.24.595788; this version posted August 19, 2024. The copyright holder for this preprint (which was not certified by peer review) is the author/funder, who has granted bioRxiv a license to display the preprint in perpetuity. It is made available under aCC-BY 4.0 International license.

Supplementary figure captions

Fig. S1. Normalized counts of the top 10 microbial species in primary tumor samples for each of 25 cancer types. The X-axis shows the species names, sorted by maximum normalized counts, measured as reads per kilobase of genome per million reads sequenced.

Supplementary table captions

Table S1. Raw counts of 5,734 TCGA WGS samples classified at the species level against the Kraken Microbial2023 database, excluding eukaryotes. The contents of Microbial2023 are described in the main text. This resulted in 11,332 species that have non-zero counts.

Table S2. Normalized counts of the values in Table S1, converted to counts per million reads sequenced.

Table S3. Normalized counts of the values in Table S2, converted to reads per kilobase of genome per million reads sequenced.

Table S4. Raw counts of 5,734 TCGA WGS samples classified at the species level against Fungi_RefSeq database. The contents of Fungi_RefSeq include all 557 fungal species in RefSeq as of late 2023.

Table S5. Normalized counts of TableS4, converted to counts per million reads sequenced.

Table S6. Normalized counts of TableS4, converted to reads per kilobase of genome per million reads sequenced.

Table S7. Raw counts of 18 selected viruses identified in each of 5,734 TCGA WGS samples by the TCGA project. The TCGA filtering process used bwa to align reads against a database including the human genome plus multiple strains of human papillomavirus (HPV), hepatitis B and C viruses (HBV, HCV-1 1, and HCV-2), and cytomegalovirus (CMV). Counts here include the 14 strains of HPV most often associated with cancer: 16, 18, 31, 33, 35, 39, 45, 51, 52, 56, 58, 59, 66, and 68. Unlike the counts from Kraken2, which treated paired reads as a unit, paired reads were counted separately here; thus if both reads in a pair aligned, this was counted as 2 matches. Note that unlike the KrakenUniq database, low-complexity sequences were not masked out prior to alignment, which might have yielded many false-positive matches for some viruses.

Table S8. TCGA study abbreviations.

Table S9. Raw counts of 4,550 TCGA WGS samples classified at the genus level against Microbial2023 database, for samples used in both this study and in the Poore et al. study. This dataset contains counts for all 3,552 genera that had non-zero counts in either study.

Table S10. Raw counts from the Poore et al. study at the genus level, for samples used in both this study and Poore et al. This set contains 4,550 samples and 3,552 genera. Raw counts included here were taken directly from Poore et al. (2020).

Table S11. Raw counts of TCGA WGS samples classified at the species level against the Fungi_RefSeq database, for samples matched with the Narunsky-Haziza et al. study. This resulted in 4,271 samples and 557 species.

bioRxiv preprint doi: https://doi.org/10.1101/2024.05.24.595788; this version posted August 19, 2024. The copyright holder for this preprint (which was not certified by peer review) is the author/funder, who has granted bioRxiv a license to display the preprint in perpetuity. It is made available under aCC-BY 4.0 International license.

Table S12. Raw counts from the Narunsky-Haziza et al. study at the species level, for samples matched with this study. This resulted in 4,271 samples and 557 species. Raw counts included here were taken directly from Narunsky-Haziza et al. (2022).

Table S13. TCGA metadata for 5,734 samples, including unique IDs for this study, IDs used by Poore et al., IDs used by Narunsky-Haziza et al., and the original TCGA identifiers.

Table S14. List of species in the Microbial2023 Kraken database, with RefSeq accessions.

Table S15. List of species in the Fungi_RefSeq Kraken database, with RefSeq accessions.

Table S16. RefSeq v200 to v220 name conversion list, for 14 of the 557 fungal species whose names were changed between the two releases of RefSeq.
